# The telomerase essential N-terminal domain promotes DNA synthesis by stabilizing short RNA–DNA hybrids

**DOI:** 10.1093/nar/gkv406

**Published:** 2015-05-04

**Authors:** Benjamin M. Akiyama, Joseph W. Parks, Michael D. Stone

**Affiliations:** 1 Department of Molecular, Cell, and Developmental Biology, University of California, Santa Cruz, CA 95064, USA; 2 Department of Chemistry and Biochemistry, University of California, Santa Cruz, CA 95064, USA; 3 Center for Molecular Biology of RNA, University of California, Santa Cruz, CA 95064, USA

## Abstract

Telomerase is an enzyme that adds repetitive DNA sequences to the ends of chromosomes and consists of two main subunits: the telomerase reverse transcriptase (TERT) protein and an associated telomerase RNA (TER). The telomerase essential N-terminal (TEN) domain is a conserved region of TERT proposed to mediate DNA substrate interactions. Here, we have employed single molecule telomerase binding assays to investigate the function of the TEN domain. Our results reveal telomeric DNA substrates bound to telomerase exhibit a dynamic equilibrium between two states: a docked conformation and an alternative conformation. The relative stabilities of the docked and alternative states correlate with the number of basepairs that can be formed between the DNA substrate and the RNA template, with more basepairing favoring the docked state. The docked state is further buttressed by the TEN domain and mutations within the TEN domain substantially alter the DNA substrate structural equilibrium. We propose a model in which the TEN domain stabilizes short RNA–DNA duplexes in the active site of the enzyme, promoting the docked state to augment telomerase processivity.

## INTRODUCTION

Telomerase is a ribonucleoprotein enzyme that maintains the ends of eukaryotic chromosomes by synthesizing repetitive DNA sequences that serve as the foundation for protective nucleoprotein structures called telomeres (1). Telomerase counteracts the loss of telomeric DNA that arises due to the inability of the conventional DNA replication machinery to completely replicate DNA ends. Thus, telomerase solves the ‘end replication problem’ and helps to avoid cell growth arrest triggered by the presence of critically short telomeres (2). Mutations within subunits of the telomerase holoenzyme give rise to genetic disorders characterized by deterioration of proliferative tissues, such as the heritable diseases dyskeratosis congenita and aplastic anemia (3). On the other hand, inappropriate telomerase activation helps to confer the ability for cells to divide indefinitely and is associated with ∼90% of human cancers, making telomerase a promising target for potential cancer therapies (4).

**Figure 1.**
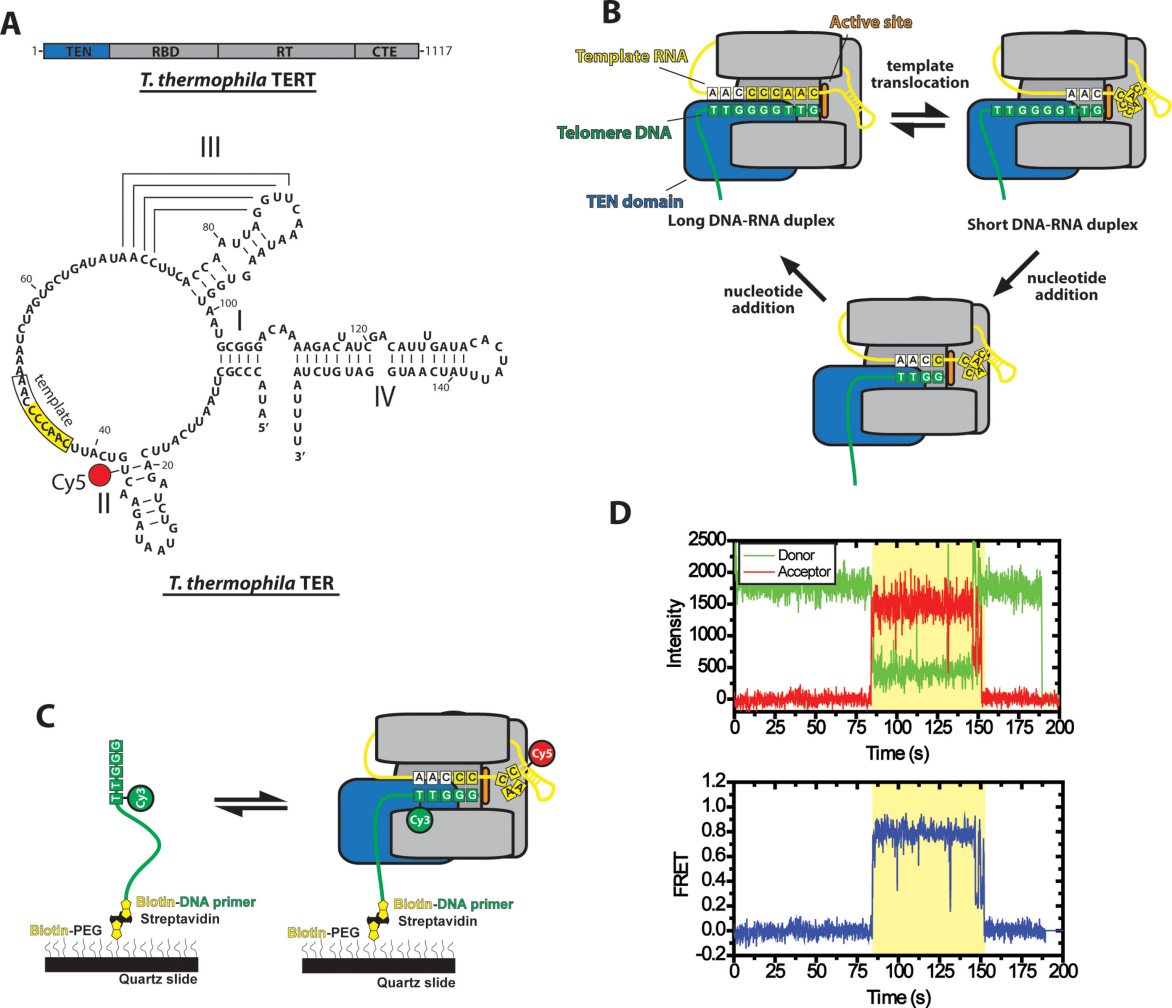
Overview of telomerase smFRET binding assay. (**A**) Domain organization of *Tetrahymena thermophila* TERT and secondary structure of *Tetrahymena thermophila* TER. TERT is divided into the telomerase essential N-terminal domain (TEN, blue), the RNA binding domain (RBD), the reverse transcriptase domain (RT) and the C-terminal extension (CTE). TER contains stems I, II, III and IV as well as a conserved RNA template (boxed region). The position of the Cy5 modification used for smFRET studies at U36 is indicated. (**B**) Diagram of telomerase catalytic cycle. TERT is represented in gray with the TEN domain highlighted in blue and the active site in orange. The telomeric DNA substrate is represented in green and the telomerase RNA is represented in yellow. The template RNA and telomere DNA form basepairing interactions and this heteroduplex is positioned in a central channel of the enzyme adjacent to the active site (6). When the end of the template is reached, the RNA–DNA duplex is denatured and the RNA template re-anneals downstream to position the template for another round of synthesis (template translocation). The post-translocation state of the enzyme contains a short RNA–DNA duplex which must be stabilized in the active site in order to become extended by the enzyme's reverse transcriptase activity to complete the catalytic cycle. (**C**) Schematic diagram of smFRET telomerase binding assay. DNA primers containing telomeric DNA sequence are labeled with a donor Cy3 dye at their 5′ most alignment residue and immobilized on a quartz microscope slide by a biotin-streptavidin linkage. Telomerase labeled with Cy5 in its TER subunit is flowed onto the slide and FRET is measured on individual molecules for the duration of the binding events. (**D**) Example smFRET trace for a (TG)_8_T_2_G_3_ primer incubated with telomerase labeled at the U36 position of the TER subunit. Donor (Cy3) and acceptor (Cy5) intensity are plotted over time (Top panel). The binding event (shaded region) is marked by the onset of a FRET signal, characterized by an anti-correlated drop in donor fluorescence and rise in acceptor fluorescence. Loss of FRET signal at ∼150s occurs either due to Cy5 photobleaching or diffusion of telomerase off of the primer. Loss of Cy3 signal at ∼190s is due to a normal process of Cy3 photobleaching. Donor and acceptor intensity values from the top panel are used to calculate a FRET trace in the bottom panel. The FRET values from each point during the binding event are combined with multiple other binding events to generate smFRET histograms.

Telomerase consists of two main components, a protein telomerase reverse transcriptase (TERT) and a telomerase RNA (TER) (Figure 1A). TERT is tightly associated with TER, and functions by repetitively reverse transcribing a short template region of TER into telomeric DNA (5). The template region basepairs with the DNA primer to form an RNA–DNA hybrid that is recognized by the TERT active site (Figure 1B) (6). The telomerase catalytic cycle can be sub-divided into two distinct activities: nucleotide addition processivity (NAP) and repeat addition processivity (RAP). During NAP, the telomere DNA substrate is progressively extended to the strictly defined template boundary. Next, during RAP the nascent DNA must dissociate from the RNA template, re-anneal downstream and enter the TERT active site for the subsequent round of NAP (5) (Figure 1B).

TERT has several conserved domains, including the telomerase N-terminal (TEN) domain, the RNA binding domain (RBD), the reverse transcriptase domain and a C-terminal extension (Figure 1A) (7). Previous studies in *Oxytricha aediculatus* have shown that TERT cross-links to a region of the telomeric DNA ∼20 nucleotides upstream of the nascent telomeric DNA 3′ end (8). Subsequently, yeast studies identified the site of a similar cross-link in the TEN domain and determined that mutations that disrupted this cross-link also had an effect on telomerase extension activity, suggesting a functional interaction (9). Studies in human telomerase also revealed an interaction site between human TERT and single-stranded DNA that occurs independently of telomerase RNA and further mapped this contact to the N-terminal region of the protein (10).

The crystal structure of the TEN domain from *Tetrahymena thermophila* revealed a protein domain with a novel fold (11). Though a co-crystal structure with DNA was not obtained, a series of surface-exposed residues were implicated in both DNA cross-linking activity and telomerase extension activity using mutagenesis experiments. A particularly interesting mutant in this study was to residue Q168, which is a highly conserved amino acid in the TEN domain found within the T2 (also known as the GQ) motif of TERT (12). A mutation to this amino acid had a severe effect on both the cross-linking activity of the enzyme and the catalytic activity of reconstituted telomerase (11). In a separate cross-linking study, a second cross-link between the DNA and the *Tetrahymena* TEN domain was identified, mapping a contact between residue W187 and the telomeric DNA primer in a region directly adjacent to the 3′ end of the DNA in the active site of the enzyme (13).

Taken together, these experiments suggested the TEN domain mediates an ‘anchor site’ interaction between the telomeric DNA substrate and the enzyme (8–9,11,13–14). The TEN anchor site model posits that the 5′ end of the DNA is bound by the TEN domain, such that when the 3′ end of the DNA dissociates from the template RNA during RAP, the anchor site interaction with the 5′ end of the DNA is maintained, preventing dissociation of the primer. Following the formulation of this model, yet another TEN domain mutant (L14A) was characterized in the *Tetrahymena* telomerase system which had a severe effect on RAP without affecting the anchor site interaction in the protein (15). Telomerase harboring a mutation to residue L14 was competent to complete a single round of telomere repeat synthesis, but could not undergo productive translocation to generate RAP products (15). Interestingly, when the L14A mutant was tested in the context of endogenous *Tetrahymena thermophila* telomerase processivity factors, the enzyme retained the ability to undergo RAP, albeit at a substantially reduced rate (16). More recently, studies on human telomerase demonstrated that the TEN domain is required for RAP and that certain TEN domain constructs could complement a TEN domain deletion mutant in *trans* to restore RAP (17).

Further studies utilizing a sensitized telomerase enzyme lacking an internal RNA template showed that human telomerase could elongate a short RNA–DNA hybrid provided in *trans* (18), and do so in a TEN domain-dependent manner (19), raising the possibility that the TEN domain may possess activities beyond providing a distal 5′ anchor site. Indeed, another study using human telomerase demonstrated that TEN domain mutants exhibit kinetic defects independent of their binding defects, suggesting that the TEN domain may have an additional function to its role as an anchor site (20).

Here, we have employed a single molecule telomerase binding assay (21,22), together with telomerase direct primer extension assays, to interrogate the impact of TEN domain mutants on telomere DNA dynamics and telomerase activity. We demonstrate that DNA substrates bound within wild-type telomerase enzymes exhibit dynamic rearrangements between two clearly resolved conformations. The observed internal DNA structural equilibrium correlates with the extent of potential hybrid formation between the DNA primer and the RNA template. Furthermore, under our experimental conditions, mutations to TEN domain residues L14, Q168, or F178 significantly alter the DNA structural equilibrium in primers with the capacity to form short RNA–DNA hybrids (< 5 bp) but not for primers that may form longer heteroduplexes in the telomerase active site. Taken together, our experiments provide evidence that a DNA primer bound within the *Tetrahymena* telomerase complex may exist in one of several possible conformations: a docked conformation wherein the DNA is hybridized to the template RNA in the active site, or a second alternative conformation in which the DNA remains bound to the enzyme but is positioned away from the active site. These results provide support for a model in which a critical function of the conserved TEN domain is to stabilize the docked conformation of the enzyme for short primers where basepairing stability is expected to be minimal.

## MATERIALS AND METHODS

### Dye-labeling of synthetic oligonucleotides

Dye-labeling of synthetic DNA and RNA fragments was performed as previously described (22,23). Synthetic DNA primers (IDT) were ordered containing an amine modification at the desired labeling site and incubated with amine-reactive Cy3 dye (GE Lifesciences) in 0.1M sodium bicarbonate solution. Synthetic RNA fragments were also ordered containing site-specific amine modifications (Dharmacon) and labeled in the same fashion. Dye-labeled oligonucleotides were purified by reverse-phase HPLC. Synthetic RNAs were then splint-ligated to generate full-length telomerase RNA, and the desired RNA was PAGE purified.

### Telomerase reconstitution and purification

Telomerase was reconstituted in rabbit reticulocyte lysate (RRL) as previously described (22). Briefly, 6 pmol of dye-labeled TER was incubated with 25 pmol of recombinant purified p65 in a final volume of 12.5 μl for 10 min at room temperature. This was added to a mixture containing 200 μl T7-coupled transcription/translation RRL (Promega), 4.13 μg FLAG-TERT expression plasmid, 5 μl PCR enhancer and 5 μl 1 mM methionine in a final volume of 250 μl. This was incubated at 30°C for 2 h. Assembled telomerase was purified by immunoprecipitation using anti-FLAG conjugated beads (Sigma). Telomerase-containing RRL was incubated overnight with anti-FLAG beads. The beads were then washed in a wash buffer containing 300 mM potassium glutamate. Telomerase was eluted in a buffer containing 1 mg/ml FLAG peptide (Sigma), 50 mM Tris pH 8.0, 1.25 mM MgCl_2_ and 10% glycerol. Aliquots of purified telomerase were flash-frozen in liquid nitrogen for future use.

### TERT mutagenesis

The FLAG-TERT expression plasmid was mutagenized using PCR mutagenesis and custom PCR primers (IDT). Linear PCR amplicons were ligated using DNA ligase (NEB) and used to transform DH5α competent cells and isolated by mini-prep (Qiagen). Each plasmid was then sequenced to determine whether it had the correct modification.

### Single-molecule FRET telomerase activity assay

Single-molecule FRET slides were thoroughly cleaned and PEGylated as described (24). Prepared slides were then incubated in 10 mg/ml BSA for 10 min, and rinsed with T50 buffer (10 mM Tris pH 8.0, 50 mM NaCl). Next, 200 μl of 10 pM purified Cy3-labeled DNA was flowed over the slide. Eluted telomerase containing a Cy5-modification in the TER subunit was added in a buffer containing 10 μl eluted telomerase, 18 μl telomerase imaging buffer (50 mM Tris pH 8.0, 1.25 mM MgCl_2_, 0.5% glucose, 10% glycerol, 1 mg/ml trolox), 1.5 μl 10 mg/ml BSA and 0.5 μl glucose-oxidase catalase solution (100 mg/ml glucose oxidase, 0.4 mg/ml catalase in T50). FRET was observed using a prism-type total internal reflection microscope on an Andor CCD camera with an integration time of 100 ms. FRET traces were analyzed using custom Matlab software (Mathworks). FRET was measured over the course of the binding event using the formula *E* = 1/(1 + γ(*I*_D_/*I*_A_)), where *E* is FRET efficiency, *I*_D_ is donor intensity and *I*_A_ is acceptor intensity. The factor γ adjusts for differences in dye quantum yields and can be useful in correcting FRET efficiency when there is a protein-induced Cy3 enhancement, as was observed in a subset of our traces. Because we cannot distinguish between acceptor bleaching events and enzyme dissociation from the primer, we could not determine γ by the previously established method (25). Instead, we approximated γ as (*I*_D1_ + *I*_A1_)/(*_I_*_D2_ + *I*_A2_), where *I*_D1_ + *I*_A1_ represents the sum of the donor and acceptor intensity before protein binding and *I*_D2_ + *I*_A2_ represents the sum of the donor and acceptor intensities after binding (22). The factor γ was determined individually for each trace and was consistent with previously reported values of protein-induced Cy3 enhancement.

### Telomerase extension assays

Telomerase for *in vitro* telomerase extension assays was prepared in RRL as described above, however instead of dye-labeled telomerase RNA, *in vitro* transcribed telomerase RNA was used. RRL reactions were not immunopurified, but were used directly in telomerase extension assays. 5 μl RRL reaction was added to1 μM DNA primer, 100 μM dTTP, 9 μM dGTP, 1 μM ^32^P α-dGTP, in a final volume of 15 μl in a buffer containing 50 mM Tris pH 8.0, 1.25 mM MgCl_2_ and 10% glycerol. Reactions were then phenol:chloroform extacted and ethanol precipitated. Prior to phenol:chloroform extraction a radiolabeled recovery control was added, consisting of 5′-end-labeled ^32^P TER. Extension products were resolved on a 12% PAGE DNA sequencing gel and imaged using a Typhoon scanner (GE Lifesciences) with a phosphor screen (GE Lifesciences).Telomerase activity assays were performed in triplicate, and gels were quantified using the program SAFA (26). The intensity of each band was corrected for the number of radio-labeled dGTPs incorporated at that band. Nucleotide addition processivity (NAP) was calculated as the sum of the first repeat addition band (defined as a DNA product extended to the 5′ end of the RNA template), plus all higher molecular weight bands in the lane. These larger products were included in the NAP calculation because they necessarily passed through the first repeat addition band intermediate. This value was then normalized using the recovery control. Experiments for wild-type or each of the mutant telomerase enzymes were analyzed by normalizing the number of primers to reach the first RAP band for each DNA primer variant to the primer with the highest RNA–DNA hybrid forming potential (GGGGTT)_3_, which was set to one_._

### RNA dot blot quantification

Telomerase was prepared in RRL and immunoprecipitated as previously described for single-molecule FRET assays, however instead of dye-labeled telomerase RNA, *in vitro* transcribed telomerase RNA was used. 2.5 μl and 5 μl aliquots of immunopurified telomerase were diluted to 10 μl in formamide loading buffer (90% deionized formamide, 0.1% bromphenol blue, 0.1% xylene cyanole and 1X TBE) and heated at 70°C for 5 min and placed on ice. The solution was dotted onto a piece of Hybond N+ membrane (GE Lifesciences) and cross-linked to the surface using a UV transilluminator for 1 min. The membrane was blocked in 10 ml Church buffer (1% BSA, 1 mM EDTA, 500 mM sodium phosphate pH 7.2, 7% SDS) at 55°C for 30 min. Approximately 3 × 10^6^ cpm of a 5′-^32^P-end-labeled DNA probe was added to the solution (sequence: 5′-TATCAGCACTAGATTTTTGGGGTTGAATG-3′) and incubated at 55°C overnight. The membrane was washed three times in 0.1X saline-sodium-citrate buffer (15 mM NaCl, 1.5 mM trisodium citrate, pH 7.0) containing 0.1% SDS at room temperature. The membrane was imaged using a phosphor screen (GE Lifesciences) and a typhoon scanner (GE lifesciences). Quantification of the blot was performed with ImageJ. To determine concentrations, samples were compared against *in vitro* transcribed telomerase RNA standards dotted onto the same blot.

### HaMMy analysis

Individual single-molecule traces were analyzed by HaMMy (27). HaMMy was instructed to identify 3 states for each trace: the 0.0 FRET unbound state, the 0.75 FRET docked state, and the 0.25 FRET alternative state for U36-labeled enzyme and the 0.0 FRET state, the 0.50 FRET state, and the 0.90 FRET state for the U63-labeled enzyme. Individual dwell times for each trace were compiled together in a single table and plotted as a histogram using Origin (Originlab). The histograms were fit to an exponential decay function y = A_0_e^−x/^τ + y_0_, where A_0_ represents the amplitude, τ represents the average dwell time and y_0_ represents the y offset.

## RESULTS

### Direct observation of DNA primer dynamics within single telomerase enzymes

To determine how the TEN domain influences conformational rearrangements within the telomerase holoeznyme, we required a method that permits direct observation of structural dynamics in telomerase bound to its DNA substrate. Traditional methods for measuring telomerase–DNA interactions cannot directly detect such dynamic structural rearrangements in a DNA primer bound within a telomerase enzyme. To overcome this challenge, we turned to a single-molecule telomerase binding assay that monitors DNA dynamics within individual telomerase-primer complexes via Förster resonance energy transfer (FRET) (21,22). The single molecule FRET (smFRET) assay provides a unique opportunity to analyze how previously characterized TEN domain mutants might alter the movement of DNA within the telomerase holoenyzme, and correlate these measurements with the effects of the mutations on telomerase activity.

In a typical experiment we analyze conformational properties of telomerase–DNA complexes using a prism-type total internal reflection fluorescence (TIRF) microscope and measure the distance-dependent energy transfer efficiency between a donor and an acceptor dye incorporated into a telomeric DNA primer and telomerase RNA (Figure 1C) (28). Unless otherwise indicated, TER was labeled with a Cy5 acceptor dye at residue U36 (Figure 1A), reconstituted into an active telomerase RNP complex and purified using a FLAG-tag engineered onto the N-terminus of TERT (22–23,29). Telomeric primers used in this study were labeled at the 5′ most alignment residue with a Cy3 dye modification. Each primer possessed a 5′-(TG)_8_ dinucleotide repeat sequence followed by varying amounts of native *Tetrahymena* telomere DNA sequence (Figure 1C). These dye modifications in either TER or the DNA primer have no detectable effect on telomerase assembly or catalysis (21,22). Furthermore, the (TG)_8_ repeat primers support wild-type telomerase activity and simplify the present experiments by ensuring the 3′-end of the DNA primer can only bind to the RNA template in a single alignment register (30).

**Figure 2.**
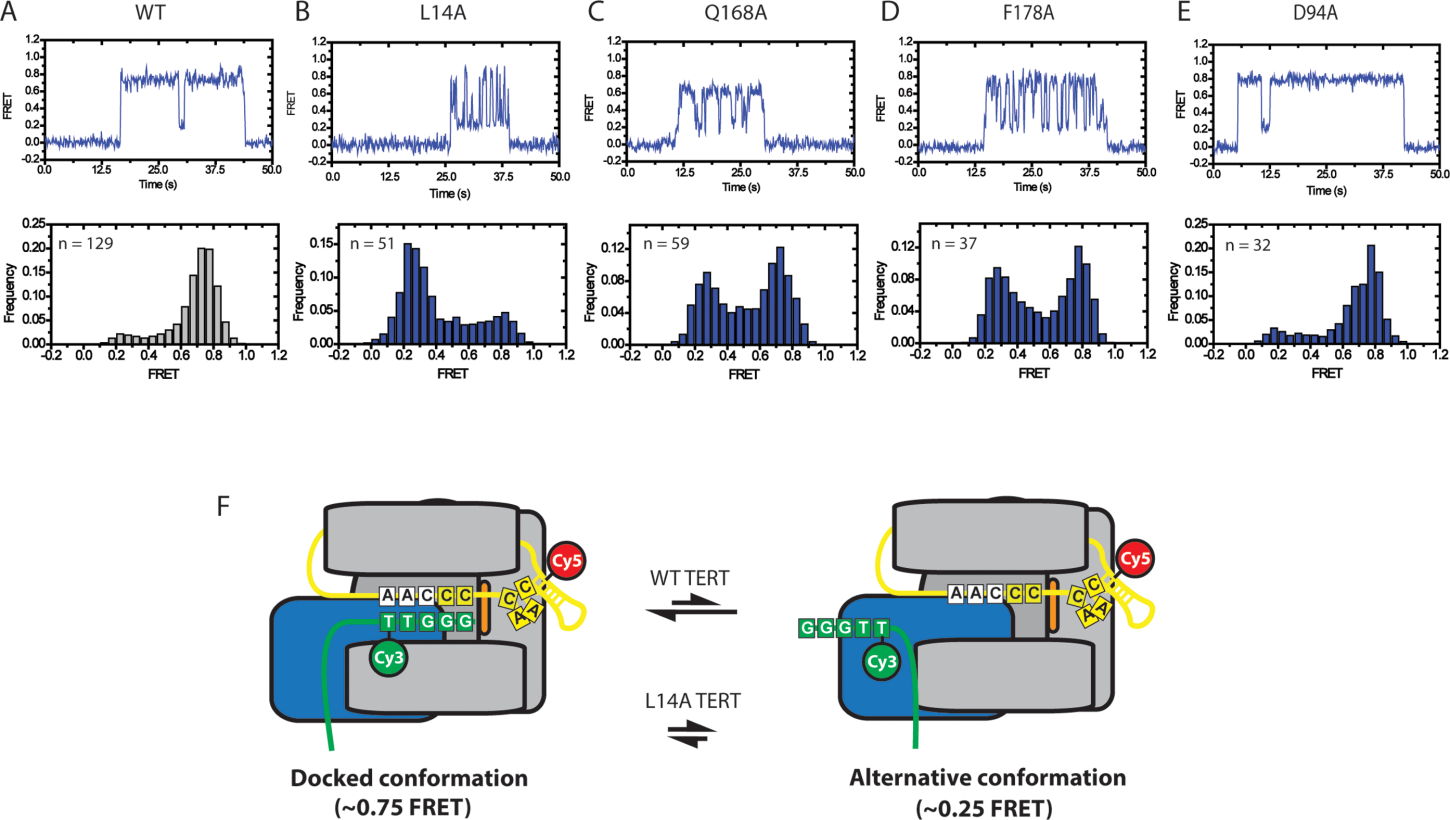
Representative smFRET traces and histograms for wild-type and mutant telomerase. (**A**) Representative smFRET trace (top) and smFRET histogram (bottom) for Cy3-labeled (TG)_8_T_2_G_3_ primer incubated with wild-type telomerase labeled with Cy5 at the U36 position of TER. Wild-type enzyme demonstrates a stable ∼0.75 FRET state with transient excursions to a ∼0.25 FRET state (top panel). This is also reflected in a smFRET histrogram of FRET values compiled from 129 separate binding events (bottom panel) demonstrating a predominant ∼0.75 FRET distribution with a small shoulder at ∼0.25 FRET. (**B**–**E**) Representative smFRET traces (top) and smFRET histograms (bottom) for L14A, Q168A and F178A mutant telomerase respectively. (**F**) Model of telomerase DNA binding dynamics. smFRET data indicate that DNA associated with telomerase can exist in one of at least two conformations. In the docked state, represented by the ∼0.75 FRET population, the RNA–DNA duplex is positioned in the enzyme active site. The ∼0.25 FRET population represents an alternative state that exists in an equilibrium with the docked state. In this conformation the 3′ end of the DNA is positioned away from the enzyme active site. TEN domain residues L14, Q168 and F178 bias the internal equilibrium towards the docked conformation. Importantly, smFRET alone does not provide sufficient information to fully map the contacts present in the alternative state. Therefore, although we can confidently assert that an alternative state exists, the schematic layout presented in this figure represents only one of many possible organizations that could comprise the alternative state of the enzyme.

To measure the interaction between telomerase and DNA substrates, Cy3-labeled DNA primers were surface-immobilized on a microscope slide followed by the addition of purified telomerase harboring Cy5-labeled TER. Binding of a Cy5-labeled telomerase enzyme to the Cy3-labeled DNA primer on the surface was observed as a sudden onset of FRET, characterized by a drop in the donor (Cy3) intensity and an increase in the acceptor (Cy5) intensity (Figure 1D, top). Raw dye intensity values were used to calculate the observed FRET efficiency (Figure 1D, bottom), defined as *FRET = I_A_/(I_A_+γI_D_)*, where *I_A_* and *I_D_* are the intensities of the acceptor and donor dyes, respectively, while γ is a correction factor used to account for effects of the local environment on the photophysical properties of the FRET dyes (25). smFRET measurements were conducted in the absence of dNTPs; thus, each experiment represents a telomerase complex bound at a different stage of the telomere repeat synthesis reaction, depending on the telomere sequence present at the 3′ end of the DNA primer. For our initial measurements, we used a (TG)_8_T_2_G_3_ primer sequence which has the capacity to form up to five basepairs of RNA–DNA hybrid when bound to telomerase (Figure 1C). We note that attempts to measure binding of primers with less telomeric sequence, (TG)_8_T_2_G or (TG)_8_T_2_G_2_, yielded very few binding events, prohibiting accurate measurements. Incubation of wild-type telomerase with (TG)_8_T_2_G_3_ primers yielded FRET trajectories that displayed a high FRET ∼0.75 state and transient excursions to a lower FRET ∼ 0.25 conformation (Figure 2A, top). When many of these binding events are compiled into a smFRET histogram the same two predominant FRET populations are observed (Figure 2A, bottom) consistent with previously reported results using the same primer and enzyme (22). These experiments demonstrate the ability of the smFRET assay to directly report on the internal structural equilibrium of a telomeric DNA primer bound to a telomerase enzyme.

### Mutations in the TEN domain alter telomere DNA dynamics

Next, we analyzed the binding properties of telomerase complexes harboring one of several single amino acid substitutions (L14A, Q168A, F178A, or D94A) in the TEN domain of TERT (11,15–16). We initially focused on the L14A substitution due to the severe RAP defect that was reported previously for this mutant (15). FRET trajectories collected on L14A telomerase–DNA complexes differed markedly from wild-type, with the low FRET ∼ 0.25 conformation becoming more populated and the high FRET ∼ 0.75 state less populated (Figure 2B). In addition, the L14A mutation gave rise to a general increase in the overall heterogeneity of the FRET behavior, as evidenced by the appearance of transient mid-FRET states in both the single molecule FRET trajectories and histograms (Figure 2B).

We next examined the binding properties of the (TG)_8_T_2_G_3_ primer to telomerase enzymes with either a Q168A or F178A mutation in the TEN domain of TERT. These two mutations were shown to reduce the efficiency of cross-linking to the 5′-end of the DNA primer and to reduce the rate of RAP in telomerase activity assays, albeit to a lesser extent than was observed with L14A mutants (11,15). When the Q168A and F178A TERT mutants were tested in our smFRET assay, a destabilization of the high FRET ∼ 0.75 state was once again observed (Figure 2C and D). However, the effect of these mutations was less pronounced than was observed with the L14A TERT mutant. Thus, it appears the degree of destabilization of the high FRET ∼ 0.75 state conferred by the L14A, Q168A and F178A mutations correlates well with the extent of the activity defects observed in telomerase direct primer extension assays. We note that the mutation to Q168A slightly down shifted the center of the ∼0.75 FRET state distribution, which may represent a slight rearrangement of this region of the DNA upon mutation of Q168.

Finally, we also investigated the binding of (TG)_8_T_2_G_3_ primers to enzymes containing a D94A mutation to the TEN domain. The crystal structure of the TEN domain indicated that D94 is positioned on the surface of the TEN domain that is distal to residues L14, Q168 and F178 (11). The D94A mutation was previously shown to have no effect on DNA cross-linking activity and to have a modest effect on telomerase extension activity (11). While RAP wasn't explicitly quantified in this study, D94A mutants display a clear banding pattern in telomerase extension assays suggesting they can perform RAP (11). In our smFRET binding assay, D94A mutants highly resemble the wild-type FRET distribution (Figure 2E and 2A), suggesting that D94 has no effect on the equilibrium between the ∼0.75 and ∼0.25 FRET states.

The dynamic FRET behavior observed in our experiments could, in principle, arise due to motions in the telomere DNA substrate, the region of TER labeled with Cy5, or both. Therefore, to investigate the physical basis for the different FRET states, we next prepared a telomerase complex reconstituted with TER labeled with Cy5 at residue U63, located within the template recognition element on the opposite side of the RNA template from residue U36 (31) (Supplementary Figure S1A). This labeling position was strategically chosen based upon previous experiments that demonstrated a FRET dye at residue U63 is well-tolerated by the enzyme (21,22). Interestingly, smFRET traces and histograms for U63-labeled wild-type telomerase bound to Cy3-labeled (TG)_8_T_2_G_3_ primer showed a reciprocal FRET signal compared to that observed for the U36-labeled telomerase enzyme, with a predominant mid FRET ∼ 0.5 state and a transient high FRET ∼ 0.9 state (compare Figure 2A and Supplementary Figure S1B). Moreover, when U63-labeled telomerase harboring the L14A mutation was incubated with (TG)_8_T_2_G_3_, the observed smFRET distribution shifted considerably, showing an increase of the FRET ∼ 0.9 state and decrease of the FRET ∼ 0.5 state (Supplementary Figure S1C). The reciprocal nature of the smFRET results from experiments with the U36- and U63-labeled telomerase is consistent with these two sites being distant from each other in three-dimensional space as suggested previously (21), and supports the notion that dynamics observed in our smFRET experiments are primarily due to movements of the DNA primer between distinct conformations, rather than RNA conformational changes.

In prior work, the ∼ 0.75 FRET state observed with U36-labeled enzyme was interpreted to represent a telomerase enzyme in which the DNA primer is hybridized to the RNA template and positioned in the active site poised for nucleotide extension (22), a conformation we will refer to as the docked state (Figure 2F). The assignment of the ∼ 0.75 FRET state to the docked conformation is further supported by our mutagenesis results, which demonstrate that mutations with known activity defects disrupt this state and the degree of disruption correlates with the known severity of the mutation. The ∼ 0.25 FRET state observed in U36-labeled enzyme represents a substantial deviation in FRET from the docked state, indicating the Cy3 label within the DNA primer has been repositioned across a length scale of several nanometers. Furthermore, the increased probability of adopting the ∼ 0.25 FRET state observed for processivity-defective mutants strongly suggests this state is not competent for telomere DNA primer extension. We therefore assign the ∼ 0.25 FRET state to a conformation in which the 3′ end of the DNA primer is displaced from the enzyme active site while the DNA remains bound to the enzyme via other contacts. However, since the smFRET experiments do not provide sufficient structural constraints to know precisely where the DNA is while in the ∼ 0.25 FRET state, we refer to this conformation as the alternative state to differentiate it from the docked state (Figure 2F). Comparing the FRET distributions from the U63-labeled enzyme (Supplementary Figure S2B) and the U36-labeled enzyme (Figure 2) strongly suggests that the ∼ 0.5 FRET state in the U63 enzyme corresponds to the docked conformation and the ∼ 0.9 FRET distribution corresponds to the alternative conformation. Finally, observation of dynamics within the smFRET traces (Figure 2) demonstrates that the two conformations are in dynamic equilibrium and the role of the TEN domain (and in particular residues L14, Q168 and F178) is to bias the equilibrium toward the docked conformation for nucleotide extension (Figure 2F arrows).

### The TEN domain stabilizes the docked conformation of the enzyme

**Figure 3.**
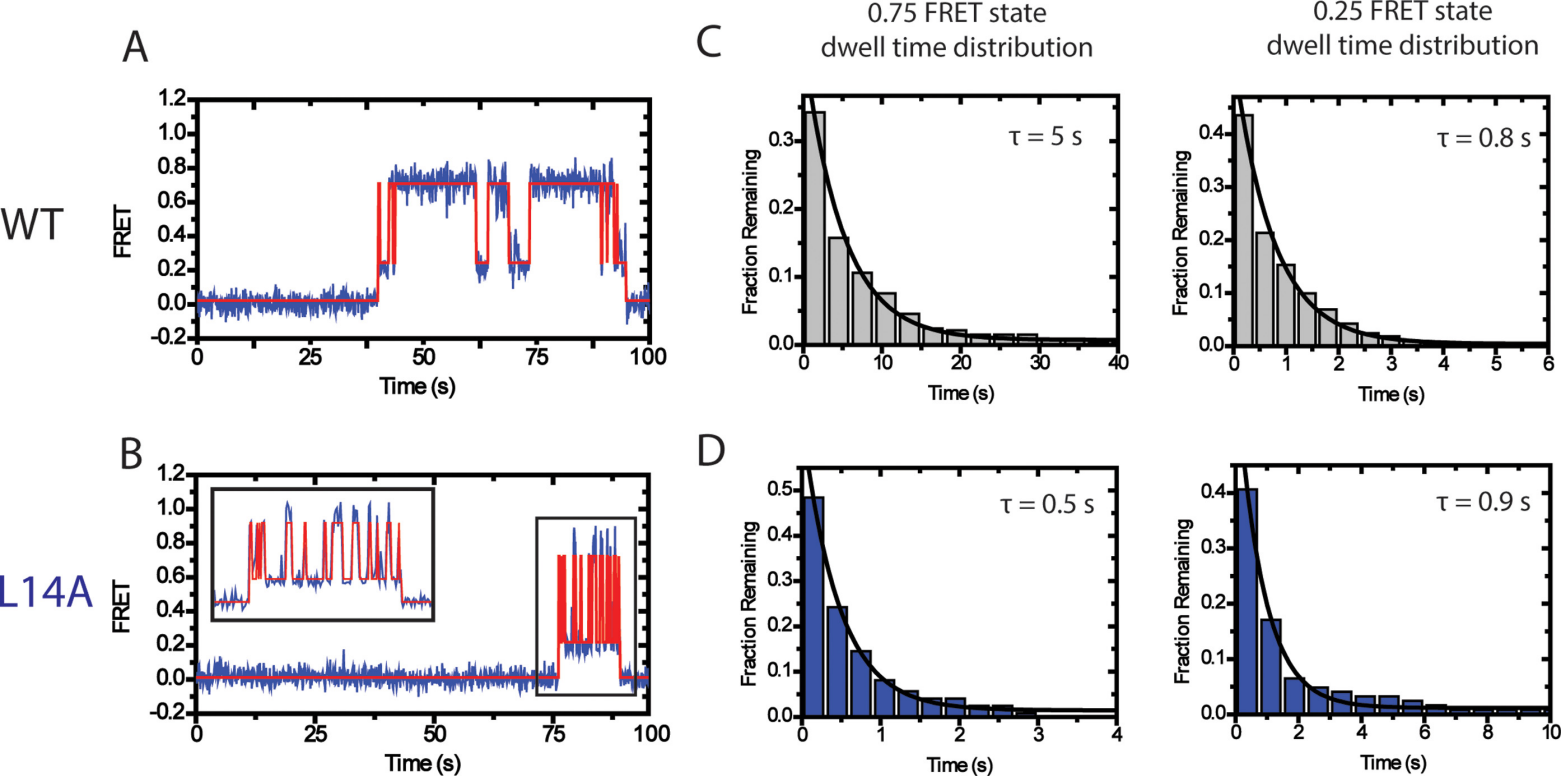
Dwell time analysis of TEN domain mutants demonstrate that the TEN domain stabilizes the docked state. (**A**) smFRET traces (blue) were analyzed by HaMMy (27) to generate idealized traces (red). These were used to determine the dwell time of the enzyme in each state. (**B**) The dwell times for the WT enzyme in the 0.75 FRET state and the 0.25 FRET state incubated with primer (TG)_8_T_2_G_3_ were compiled into histograms. The histograms were fit to an exponential function to identify the average dwell time. Wild-type TERT demonstrated a dwell time of τ_docked_ = 5 s for the 0.75 FRET state and a dwell time of τ_alt_ = 0.8 s for the 0.25 FRET state. (**C**) Representative smFRET trace and idealized HaMMy trace for L14A TERT telomerase incubated with the (TG)_8_T_2_G_3_ primer. (**D**) Compiled histograms for L14A enzyme. L14A TERT demonstrated a dwell time of τ_docked_ = 0.5 s for the 0.75 FRET state and a dwell time of τ_alt_ = 0.9 s for the 0.25 FRET state.

Importantly, the analysis of smFRET histograms alone cannot determine whether mutations to the TEN domain destabilize the docked state of the enzyme, or if these mutations bias the internal DNA equilibrium by stabilizing the alternative state of the enzyme. Therefore, to distinguish between these two possibilities we used a hidden-Markov modeling software program that generates idealized FRET trajectories for each of the telomerase–DNA binding events (Figure 3A and B) (27). To simplify the analysis, we elected to treat the internal structural equilibrium of the DNA primer as a two-state system between the low and high FRET states observed in the data collected on the wild-type and L14A enzymes (Figure 2A and B). The idealized FRET trajectories were then used to generate dwell time distributions of the time spent in either the high FRET docked state or low FRET alternative state (Figure 3C and D). Each dwell time distribution contained at least 100 individual dwell time measurements and was well fit by a single exponential decay function. For wild-type telomerase bound to the (TG)_8_T_2_G_3_ DNA primer, the average time spent in the high FRET docked conformation (τ_docked_) was 5 s while the alternative conformation dwell time (τ_alt_) was 0.8 s (Figure 3C). By comparison, when L14A mutant telomerase was incubated with the (TG)_8_T_2_G_3_ primer, the average dwell time for the high FRET docked conformation dropped by an order of magnitude to 0.5 s, whereas the dwell time for the low FRET alternative state remained essentially unchanged at 0.9 s (Figure 3D). As a control, we performed the same kinetic analysis using data collected on U63-labeled wild-type and L14A mutant telomerase enzymes bound to the (TG)_8_T_2_G_3_ primer. In this case, the L14A mutation exerted the largest effect on the dwell time distribution of the predominant FRET ∼ 0.5 state, corresponding to the docked conformation (Supplementary Figure S2A–D). Since a mutation to L14 destabilizes the docked conformation but has a negligible impact on the stability of the alternative conformation, we conclude the TEN domain stabilizes the docked conformation of the DNA primer.

In principle, the TEN domain mutations analyzed in our smFRET experiments could also impact the overall binding lifetimes of the DNA primer to telomerase. Qualitative inspection of the binding data for the wild-type and mutant enzymes indicates that all enzymes are competent to bind to the DNA primer on similar timescales (Figure 2). However, it is important to note that under the conditions of our assay we cannot readily distinguish between enzyme dissociation and photobleaching of the acceptor dye, precluding accurate determination of enzyme off-rates.

### The TEN domain stabilizes short RNA–DNA duplexes

**Figure 4.**
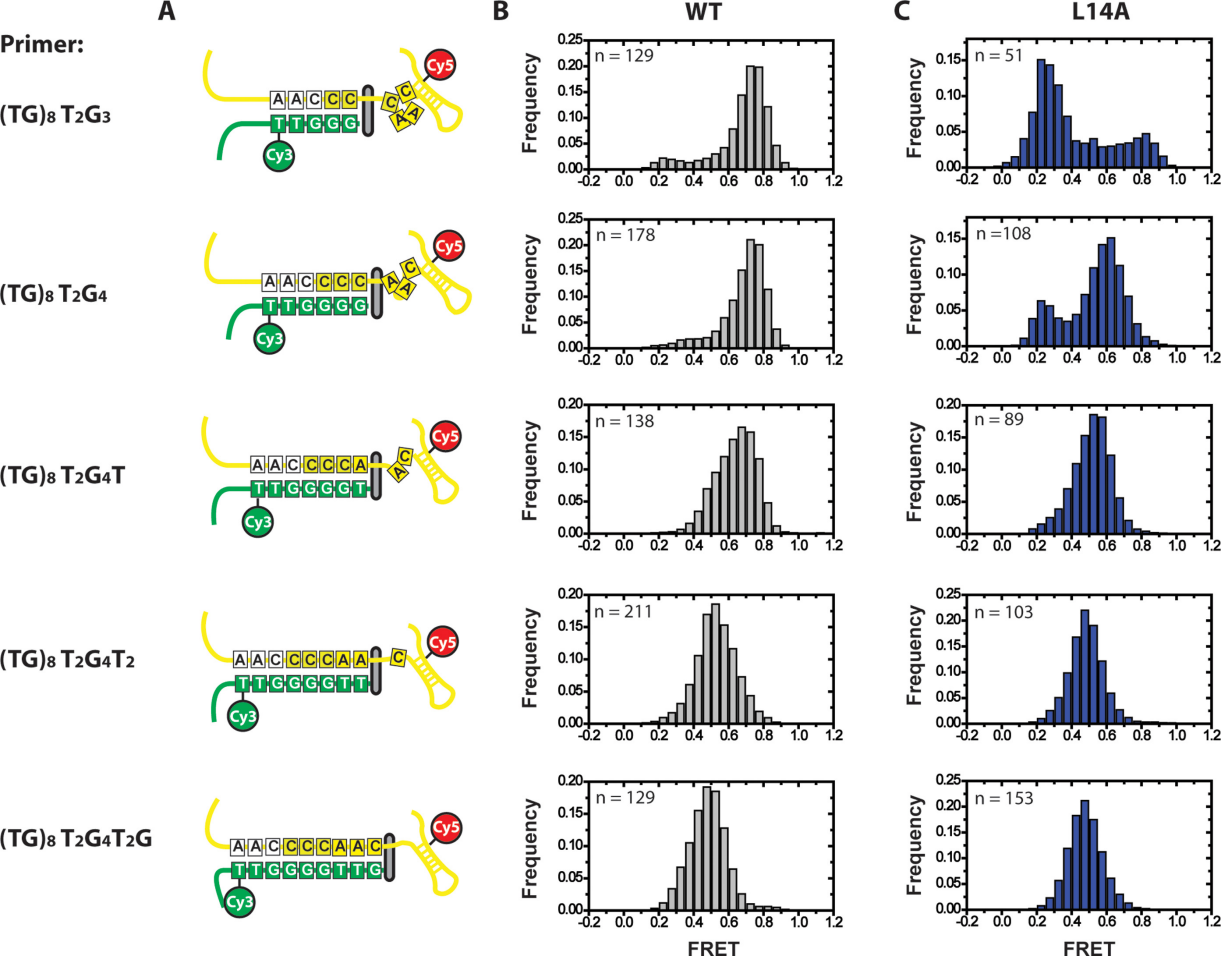
Effect of primer-template hybrid formation on FRET distributions. Primers capable of forming 5–9 basepairs with template RNA were tested in smFRET telomerase binding assays with U36-labeled telomerase. (**A**) Schematic diagram of the docked state for all six primers used in smFRET experiments, demonstrating the number of basepairs formed and the expansion of the template RNA as the RNA–DNA duplex becomes progressively longer (22). (**B**) smFRET histograms for wild-type enzyme. As primers contain progressively more telomeric DNA sequence, the predominant FRET distribution of the docked state shifts from ∼0.75 FRET to ∼0.5 FRET. In addition, the ∼0.25 FRET alternative state disappears. (**C**) smFRET histograms for L14A mutant enzyme.

Having identified a critical role for the TEN domain in stabilizing the docked conformation for the (TG)_8_T_2_G_3_ primer, we next set out to analyze the effect of varying the amount of telomeric DNA sequence in the primer. We repeated the smFRET telomerase binding experiments with a set of DNA primers, each having one additional nucleotide of telomeric sequence added at the 3′-end. Therefore, these primers can in principle make increasing numbers of basepairing contacts with the template region of TER, with the (TG)_8_T_2_G_3_ primer having the potential to make a five basepair RNA–DNA duplex and the (TG)_8_T_2_G_4_T_2_G primer having the capacity to form up to nine basepairs of RNA–DNA duplex (Figure 4A).

As primers with increasing amounts of telomeric sequence are used, the wild-type FRET distribution undergoes two main changes. First, the predominant FRET state, which we have assigned to the docked conformation, undergoes a gradual shift from a distribution centered at ∼0.75 FRET to a distribution centered at ∼0.5 FRET (Figure 4B). This drop in FRET was previously demonstrated to represent an expansion in the flexible region of RNA 5′ of the template as the RNA–DNA duplex is extruded from the active site (22), consistent with the notion that the high FRET state is reporting on the docked conformation of the DNA primer. The second trend observed is that the ∼0.25 FRET distribution disappears in smFRET histograms for primers with increasing telomeric DNA sequence. A likely explanation for this observation is that later catalytic intermediates possess greater numbers of RNA–DNA basepairs which would be expected to stabilize the docked conformation of the enzyme at the expense of the alternative conformation.

Next, we tested L14A mutant telomerase with primers possessing increasing amounts of telomeric sequence. Interestingly, L14A mutant telomerase showed only a modest defect in formation of the high FRET docked conformation with the (TG)_8_T_2_G_4_ primer, which has the capacity to form one additional basepair with the TER template region when compared with the (TG)_8_T_2_G_3_ primer (compare Figure 4B and C). As the amount of telomeric sequence was further increased, the DNA binding properties of the L14A mutant enzyme resembled the wild-type distributions, with no detectable difference in smFRET distributions observed for the (TG)_8_T_2_G_4_T_2_G primer, which can form up to nine basepairs of RNA–DNA duplex (Figure 4B and C). A similar result was obtained when the same set of experiments was performed U63-labeled telomerase, only with the expected inversion of the high and low FRET states as described earlier (Supplementary Figure S3A–C). In addition, the same correlation between increased occupancy of the transient alternative DNA conformation with DNA primers possessing less telomeric sequence was evident for the Q168A and F178A mutants (Supplementary Figure S4A–D). We note that in addition to impacting the dynamic equilibrium of the docked and alternative states, the L14A mutant also has a detectable impact on the centers of the FRET distributions of the docked conformation with the (TG)_8_T_2_G_4_ and (TG)_8_T_2_G_4_T primers, which may be due to a slight difference in the structure of the docked state when compared with the wild-type enzyme. Taken together, our results demonstrate the L14A, Q168A and F178A mutants exhibit detectable defects in early catalytic intermediates that contain short RNA–DNA duplexes; however, this DNA binding defect is suppressed for primers corresponding to late catalytic intermediates with the potential to form long RNA–DNA duplexes.

### TEN domain mutants fail to extend primers with low RNA–DNA hybrid potential

The telomerase catalytic cycle is often sub-divided into two separate stages: nucleotide addition processivity (NAP) and repeat addition processivity (RAP). NAP is typically used to describe the synthesis of one telomeric DNA repeat, while RAP refers to the series of molecular rearrangements required to realign the telomerase RNA and telomere DNA subunits in order to add additional telomeric repeats. RAP involves several steps including: pausing nucleotide extension correctly at the end of one telomeric repeat, melting of the existing RNA–DNA duplex, reannealing of a short 3 basepair RNA–DNA duplex in the next alignment register, and extension of the newly-formed short RNA–DNA duplex (Summarized in Figure 1B). Our data suggest that mutations to L14, Q168 and F178 impact this final step in RAP, by preventing the stable association of short RNA–DNA duplexes in the enzyme active site. If this interpretation is true, we would anticipate that L14A, Q168A and F178A mutants would not only manifest themselves as RAP defective mutants, but should also exhibit NAP defects for primers that form short RNA–DNA duplexes.

**Figure 5.**
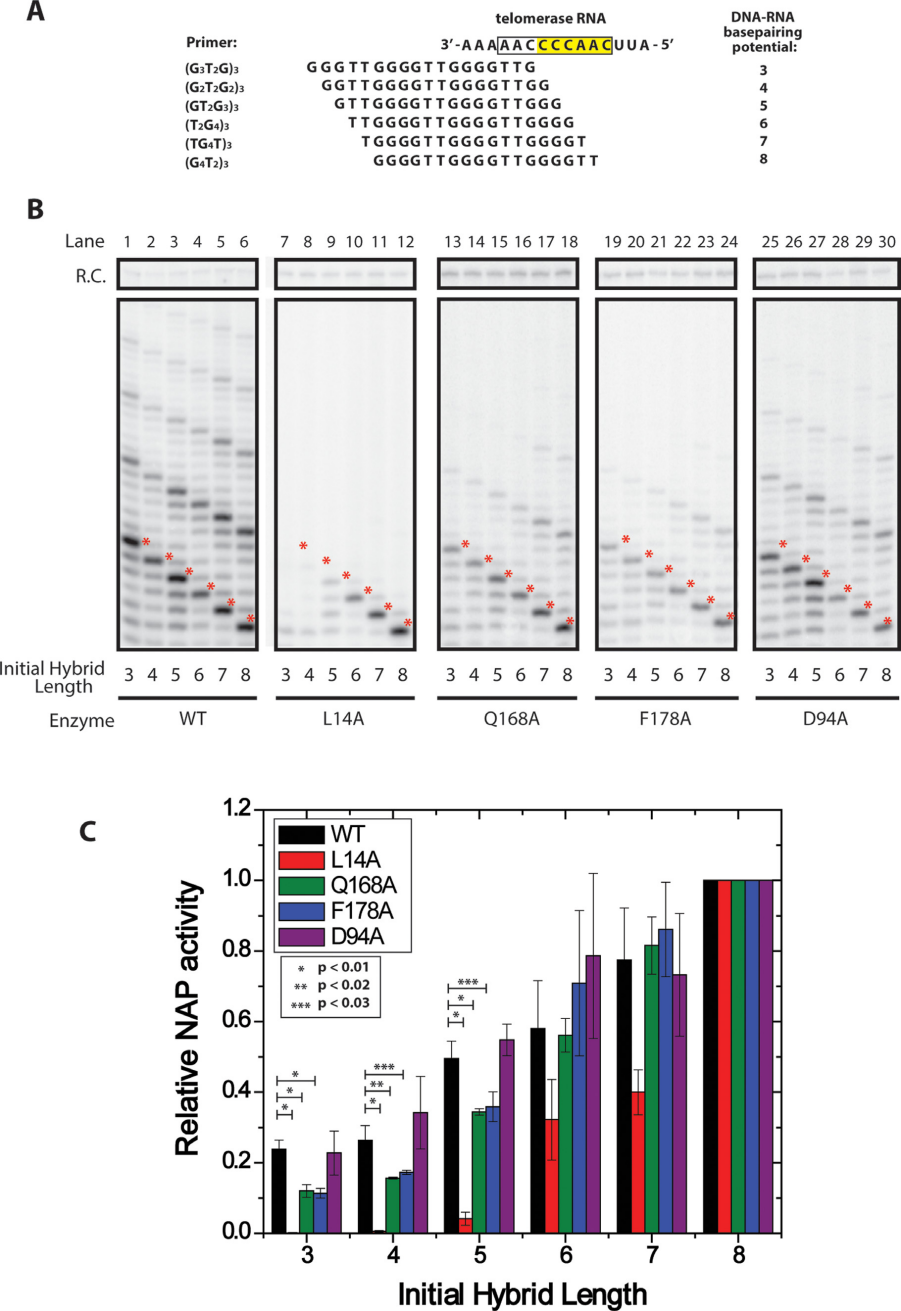
Telomerase activity assays demonstrate TEN domain mutations affect nucleotide addition processivity of primers with short RNA–DNA duplexes. (**A**) Primer permutants used in *in vitro* extension assays. Primers were length-matched at 3 telomeric repeats (18 nts), but staggered such that they formed different initial potential RNA–DNA duplex lengths with template RNA. (**B**) Telomerase was reconstituted in rabbit reticulocyte lysate and telomerase activity was assayed on the six DNA primers corresponding to six potential RNA–DNA hybrid lengths. WT enzyme was compared against enzyme harboring L14A, Q168A, F178A and D94A mutations. Mutants were assayed for primer-specific NAP defects by comparing the accumulation of the first repeat addition band (red asterisks) between primers tested with the same enzyme. (**C**) Quantification of relative NAP activity as a function of initial primer duplex length. Telomerase activity assay gels (Figure 5B) were performed in triplicate and quantified using the program SAFA (26). The quantification of each band was then corrected for specific activity. NAP was quantified as the amount of product that was extended to the 5′ end of the RNA template (Figure 5B, red asterisks, see Materials and Methods for details). For each wild-type or mutant enzyme, the observed NAP activity for each primer variant was normalized to the (GGGGTT)_3_ primer, which has the potential to form eight basepairs of RNA–DNA hybrid, and displayed the maximal NAP activity. P-values indicating statistical significance are as marked on the graph, error bars indicate one standard deviation based on triplicate measurements.

To test this prediction, we performed direct primer extension assays with either wild-type TERT or TERT bearing mutations in the TEN domain (L14A, Q168A, F178A, or D94A) using a set of DNA primers that were all 18 nucleotides in length, but had staggered sequences that permitted formation of varying amounts of RNA–DNA hybrid in the telomerase active site (Figure 5A). As expected, wild-type telomerase efficiently extended all six primers to the first complete telomeric DNA repeat band (Figure 5B, red asterisks) and exhibited RAP as evidenced by the accumulation of products extended by multiple telomere repeats. In contrast, telomerase enzymes harboring the L14A mutation were severely perturbed in NAP for primers with low RNA–DNA duplex potential, but as RNA–DNA hybrid potential increased NAP activity was restored (Figure 5B, compare lanes 7–9 with 10–12), in close agreement with previous activity assays on the L14A enzyme (15). Moreover, primers that were successfully extended by the L14A mutant telomerase to the end of the first nascent telomeric DNA repeat failed to extend beyond this point. This result demonstrates that the RAP defect observed in the L14A mutant enzyme is due to the inability of this enzyme to position short RNA–DNA hybrids in the active site and is consistent with our smFRET observations. The Q168A and F178A mutants also displayed a bias in their extension activity with respect to RNA–DNA duplex potential; however the defect was not as severe when compared to the L14A mutant (Figure 5B, compare lanes 7–9, 13–15 and 19–21). The D94A mutant, which demonstrated no detectable defect in our smFRET assays (Figure 2E), similarly did not display a bias against short primers in primer extension assays (Figure 5B, compare lanes 1–3 and 25–27).

We quantified each enzyme's primer-dependent NAP activity by measuring the relative number of primers extended to the 5′ end of the telomerase RNA template (Figure 5B, red asterisks) as a function of the initial RNA–DNA duplex length (Figure 5C, see Materials and Methods for details). Data for the DNA primer variants extended by wild-type and each mutant enzyme were normalized to the activity observed for the primer with the greatest initial RNA–DNA hybrid potential [(GGGGTT)_3_], which was set to a value of one. In this way our analysis directly compares the relative efficiency of extension for primers with short initial RNA–DNA duplexes to that of primers with longer initial RNA–DNA duplexes for a particular enzyme variant.

When corrected for specific activity, our results demonstrate that even wild-type enzyme extends significantly fewer primers to their first repeat addition band when primers contain short initial RNA–DNA duplex lengths versus when primers contain long RNA–DNA duplexes (Figure 5C, black bars) This defect for short initial duplexes is shown to be significantly exaggerated in many of the TEN domain mutants (L14A, Q168A and F178A) (Figure 5C). For example, a primer initially containing a 3 basepair RNA–DNA duplex displays 24% of maximal NAP activity for wild-type enzyme; however L14A enzyme exhibited less than 1% maximal NAP activity, while the Q178A and F178A mutants each exhibited 9% maximal NAP activity. Importantly, as the initial RNA–DNA duplex length of the primers was extended, the TEN domain mutants increasingly resembled wild-type levels of activity, consistent with our smFRET measurements with primers possessing greater RNA–DNA hybrid potential. D94A mutants, which demonstrated no detectible defect in smFRET assays, closely resembled wild-type enzyme in their extension activity with all of the primers (Figure 5C). These results reveal that conditions that favor the alternative DNA conformation as measured in the smFRET assays (Figure 4) (ie. primers with low RNA–DNA hybrid potential or TEN domain mutations) also manifest as NAP defects in the direct primer extension assays (Figure 5).

In addition to the impact we observed on NAP activity, it is possible that the TEN domain mutants used in our study may negatively impact protein expression, stability, or telomerase RNA association. We note that our telomerase activity assays were internally controlled for this possibility by comparing the activity of telomerase on different primers within the same enzyme preparation. However, to determine whether any of the TEN domain mutants altered telomerase RNP assembly in our reconstitution system, we performed filter binding assays. Telomerase complexes were immunopurified from rabbit reticulocyte lysate to measure the amount of RNA assembled with TERT in the context of TEN domain mutants (Supplementary Figure S5A). The results demonstrated that most TEN domain mutants assemble a similar amount of TERT-TER RNP complexes, with only the Q168A mutant demonstrating a slightly reduced amount of protein-RNA complex. Taken together, these results suggest that these mutations do not act at the level of protein stability or protein-RNA assembly. Furthermore, the material used in the filter binding assays displayed the same RAP defects for the L14A, Q168A and F178A mutants (Supplementary Figure S5B).

## DISCUSSION

**Figure 6.**
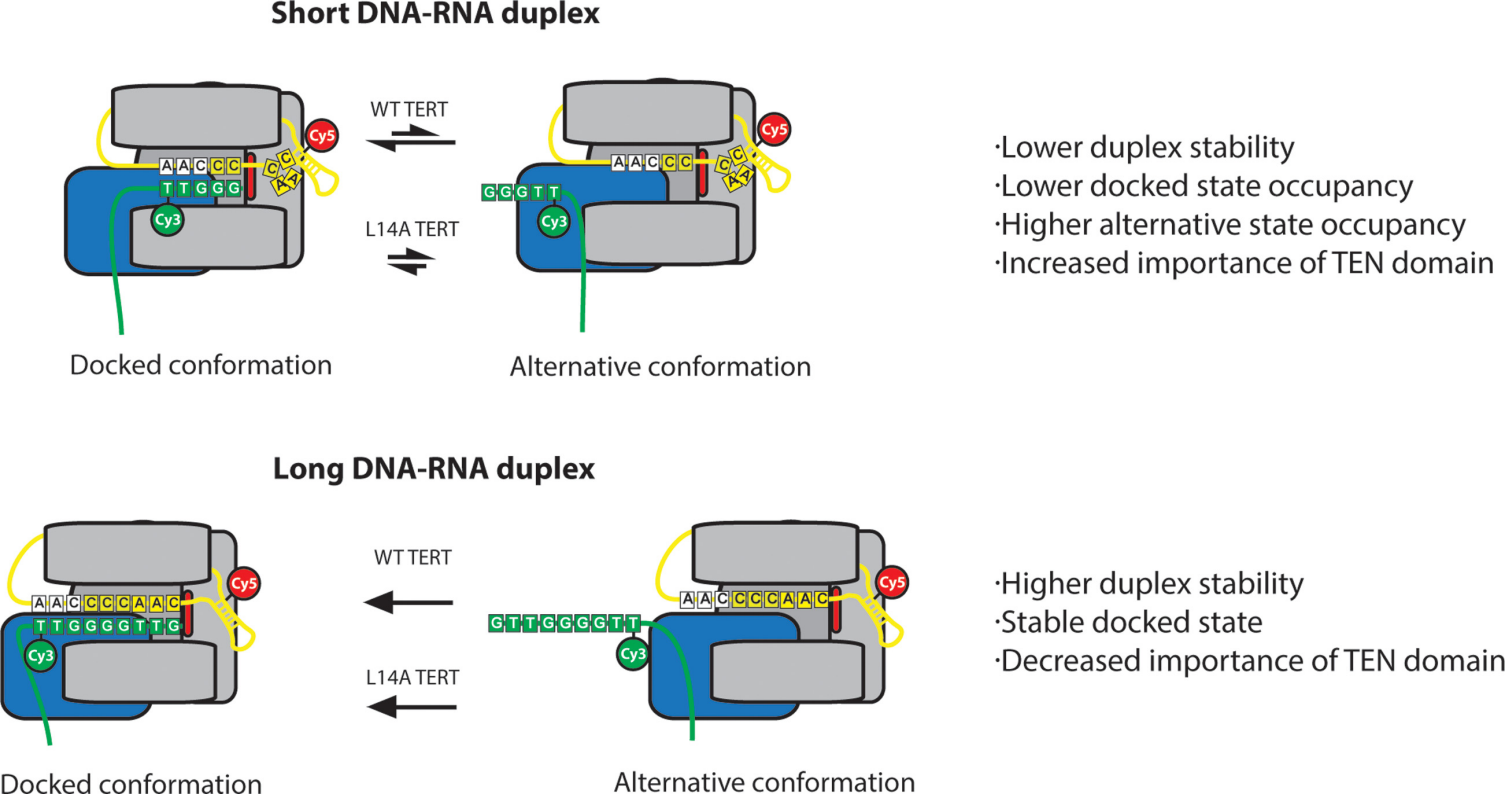
Model demonstrating the role of the TEN domain in stabilizing short RNA–DNA duplexes. Primers corresponding to early catalytic intermediates that contain fewer RNA–DNA basepairs are in a conformational equilibrium between a docked state and an alternative state (top panel). TEN domain mutants L14A, Q168A and F178A destabilize the docked state such that the alternative state is favored, disrupting the catalytic activity of the enzyme. In primers corresponding to late catalytic intermediates, the docked state is heavily favored due to the presence of additional RNA–DNA basepairs (bottom panel). As a result, the alternative state is not observed, even in the presence of TEN domain mutants.

Previous experiments investigating the role of the TEN domain established the TEN domain as an important site of DNA interaction and identified TEN domain mutants that severely affect the rate of RAP (8–9,11,15). Here, we conducted smFRET assays to investigate in real-time the effect of TEN domain mutants on DNA dynamics within the telomerase holoenzyme. These assays revealed that telomerase bound to a DNA primer exists in two distinct conformations that are in dynamic equilibrium (Figure 2). We assigned these two conformations to a docked state of the enzyme and an alternative state in which the 3′ end of the DNA is displaced from the enzyme active site (Figure 2F). Several lines of evidence support this model. First, there is a strong correlation between the relative occupancies of the alternative and docked states and the strength of processivity defects observed in TEN domain mutants in our telomerase extension assays (Figures 2 and 5). This is highly consistent with the interpretation that the docked state contains DNA positioned in the active site and the alternative state contains DNA positioned away from the active site. The correlation between activity and the docked state occupancy extends not only to several different mutations but also extends across several DNA primers, such that long RNA–DNA duplex primers that favor the docked state demonstrate a reduced sensitivity to TEN mutations (Figures 4 and 5). Finally, the model that the docked state is stabilized by template-product basepairing is further supported by our smFRET results, as primers with low RNA–DNA hybrid potential demonstrate increased primer dynamics, increased occupancy of the alternative state, and increased susceptibility to TEN domain mutations (Figures 4 and 5). We conclude that an essential role of the TEN domain in *Tetrahymena* TERT is to stabilize the short RNA–DNA duplex in the active site of the enzyme at the start of each telomere repeat synthesis cycle (Figure 6).

Our results also explain how telomerase accessory factors can partially rescue TEN domain mutants as previously observed (16). In our smFRET observations, TEN domain mutants retain the ability to enter the docked state, but do not remain in the docked state stably enough for nucleotide extension. Accessory factors which tether the DNA to the enzyme and lower the substrate off-rate would permit the DNA to remain bound for a sufficiently long time such that the enzyme has an increased probability of stably entering the docked state by chance, permitting multiple rounds of RAP to occur in these complexes, albeit at a reduced rate.

While our smFRET results demonstrate an unambiguous equilibrium between the docked and alternative states, the mechanistic and structural details of these two states remain unclear. For this reason, we do not yet know the exact mechanism by which L14, Q168 and F178 stabilize short RNA–DNA duplexes. Interestingly, residues Q168 and F178 were previously implicated in TEN domain DNA binding by mutagenesis and binding studies suggesting they may interact directly with telomeric DNA (11). Glutamine and phenylalanine also contain functional groups that can form hydrogen bonding and base stacking interactions with DNA, respectively. This suggests a possible mechanism of DNA interaction.

On the other hand, L14 was not implicated in direct DNA interactions by mutagenesis studies (15). In the crystal structure, L14 is surface exposed and makes interactions with several other hydrophobic side chains near the surface of the domain (11). L14 therefore may be important in protein–protein interactions, which may either help organize an adjacent region of TEN within the domain, or alternatively interact with another domain of TERT to aid in the positioning and/or dynamics of the TEN domain within the context of full-length TERT as suggested previously (15). Future experiments designed to directly interrogate movements of the TEN domain during telomerase catalysis will be necessary to support or refute these models.

The conservation of the TEN domain across species—including *Tetrahymena*, *S. cerevisae*, and human telomerase—suggests that the role of the TEN domain in stabilizing short RNA–DNA hybrids may be evolutionarily conserved. A conserved role for the TEN domain is further supported by a recent report that demonstrated a sensitized human telomerase enzyme, lacking both an internal RNA template and the TEN domain, can only extend a short RNA–DNA hybrid *in trans* if the TEN domain is added as a separately folded polypeptide (19). Another argument for the conserved role of the TEN domain is the incredible degree of conservation observed between species for the residue Q168. This residue is found in a region of high conservation in both yeast and human telomerases and recently it was demonstrated that a mutation to the equivalent Q169 residue in human TERT had a similar defect in RAP in human telomerase (32). Since this glutamine is conserved between *T. thermophila* and humans, and mutations to this glutamine have analogous defects in *T. thermophila* and humans, it is likely that the residue acts by a similar mechanism in the two systems, namely by stabilizing short RNA–DNA duplexes in the telomerase active site.

The conservation of L14 between *T. thermophila* and human telomerase is less clear, however previous experiments demonstrated that a double mutation to leucines 13 and 14 in human telomerase has a severe activity defect, including at least a modest defect in RAP (15). Residue F178 in *T. thermophila* telomerase does not appear to be strongly conserved, and it is less clear if it has an analogue in the human system. Nevertheless, when one considers the conservation of the other two TEN residues involved in the stabilization of short RNA–DNA duplexes and the similar activity defects observed between TEN mutants in *T. thermophila* and human telomerases, it appears that the mechanism of the TEN domain is likely conserved between species.

A recent smFRET study on human telomerase revealed a DNA dynamic equilibrium between two separate template annealing registers—pre- and post-translocation—for DNA primers corresponding to late catalytic intermediates (33). However, DNA conformational changes in early catalytic intermediates corresponding to an analogous alternative state to the one described in the present work on *Tetrahymena* telomerase were not observed. It is possible that the distinct number of alignment residues in the *Tetrahymena* and human telomerase RNA templates may confer different levels of stability to the realigned RNA–DNA hybrid at the start of each NAP cycle. Therefore, the human enzyme may require TEN domain mutants to sufficiently destabilize the docked state in order to reveal an equilibrium between a docked state and an alternative state. Future experiments comparing FRET distributions between wild-type and TEN mutant enzymes in the human enzyme will be valuable to determine if the TEN domain plays a conserved role in stabilization of short RNA–DNA duplexes in other telomerase systems.

## SUPPLEMENTARY DATA

Supplementary Data are available at NAR Online.

SUPPLEMENTARY DATA
