# Supplementary material for: The telomerase essential N-terminal domain promotes DNA synthesis by stabilizing short RNA–DNA hybrids
Source: Nucleic Acids Res. 2015 May 4;43(11):5537–49. doi: 10.1093/nar/gkv406 (PMC4477650; doi:10.1093/nar/gkv406)
Supplement: SUPPLEMENTARY DATA [file supp_43_11_5537__index.html]

The telomerase essential N-terminal domain promotes DNA synthesis by stabilizing short RNA–DNA hybrids — The telomerase essential N-terminal domain promotes DNA synthesis by stabilizing short RNA–DNA hybrids — SUPPLEMENTARY DATA 

# The telomerase essential N-terminal domain promotes DNA synthesis by stabilizing short RNA–DNA hybrids

## SUPPLEMENTARY DATA

**Files in this Data Supplement:**

- SUPPLEMENTARY DATA
