# Supplementary material for: The telomerase essential N-terminal domain promotes DNA synthesis by stabilizing short RNA–DNA hybrids
Source: Nucleic Acids Res. 2015 May 4;43(11):5537–49. doi: 10.1093/nar/gkv406 (PMC4477650; doi:10.1093/nar/gkv406)
Supplement: SUPPLEMENTARY DATA [file supp_gkv406_nar-03400-y-2014-File008.pdf]

Supplementary Information

The telomerase essential N-terminal domain promotes DNA synthesis by stabilizing short RNA-DNA hybrids

Benjamin M. Akiyama, Joseph W. Parks, and Michael D. Stone

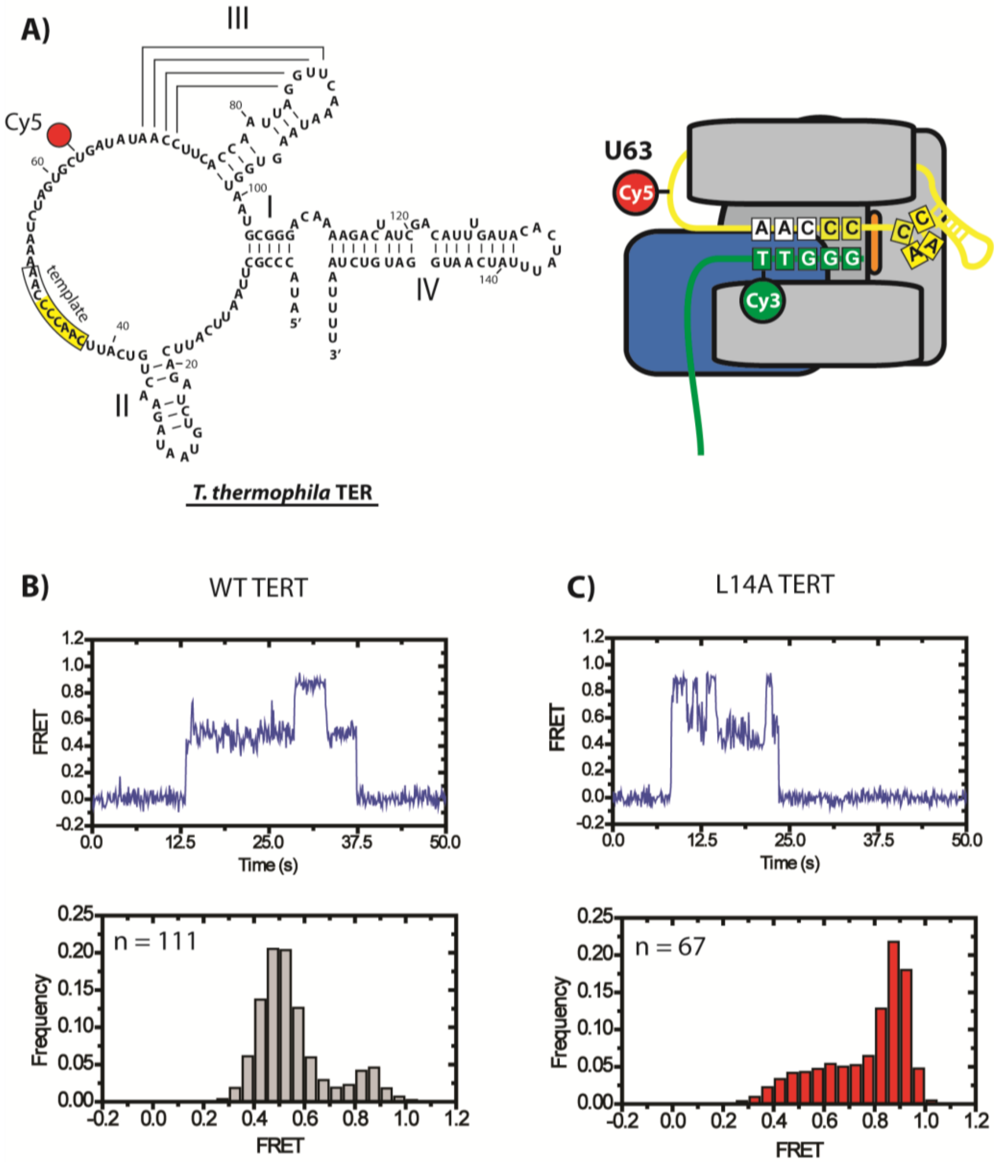

Figure S1. Telomerase labeled with an alternative labeling site demonstrates a similar distribution of states. A) (Left panel) Secondary structure of *Tetrahymena thermophila* TER with the position of the U63 Cy5 modification indicated. (Right panel) Cartoon model of the relative positions of the U63 Cy5 label and primer Cy3 label within the telomerase holoenzyme. **B)** Wild-type TERT was reconstituted with U63-labeled TER and tested in smFRET binding assays with $(TG)_8T_2G_3$ primers as in Figure 2. The top panel demonstrates a representative single molecule trace and the bottom panel represents a histogram compiled from 111 separate binding events. Both single molecule traces and histograms demonstrate a major population at ~ 0.50 FRET and a minor population at ~ 0.90 FRET. **C)** Representative single molecule trace (top) and smFRET histogram (bottom) for L14A TERT reconstituted with U63-labeled TER and Cy3-labeled $(TG)_8T_2G_3$ primers.

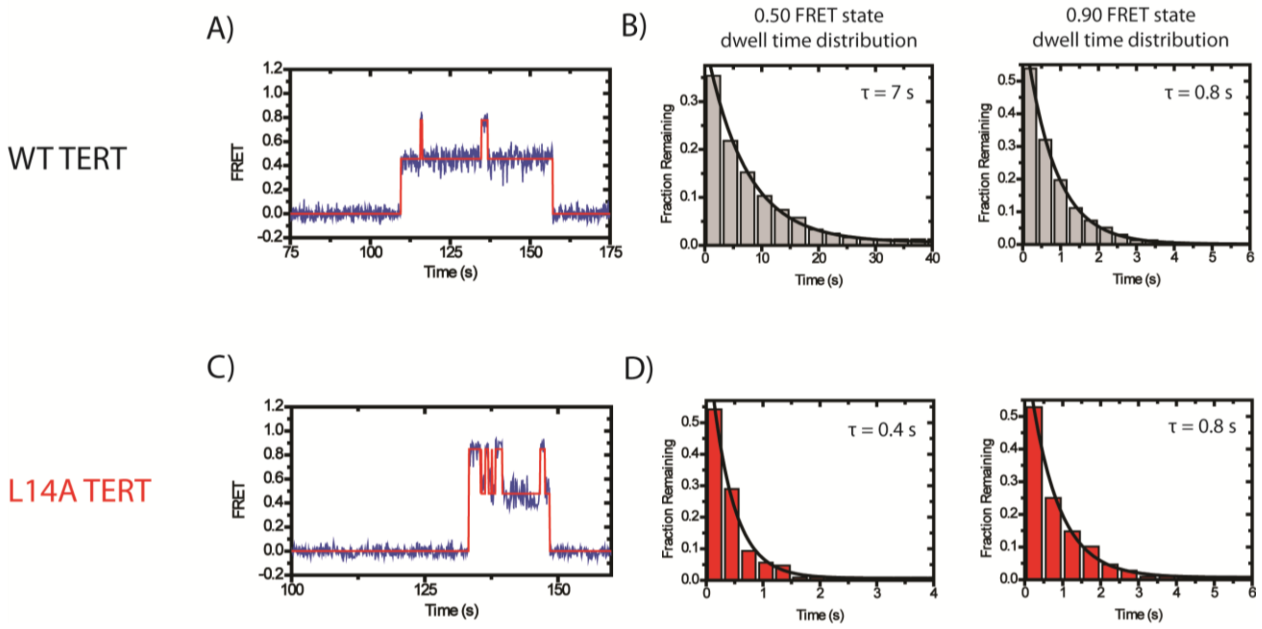

Figure S2. Dwell-time analysis using U63-labeled telomerase. A) U63-labeled TER was reconstituted with wild-type TERT and tested in the smFRET binding assay using $(TG)_8T_2G_3$ primers. smFRET traces (blue) were analyzed by HaMMy (1) to generate idealized traces (red). These were used to determine the dwell time of the enzyme in each state. **B)** The dwell times for the wild-type enzyme in the 0.50 FRET state and the 0.90 FRET state incubated with primer $(TG)_8T_2G_3$ were compiled into histograms. The histograms were fit to an exponential function to identify the average dwell time. Wild-type TERT demonstrated a dwell time of τ_{docked} = 7 s for the 0.50 FRET state and a dwell time of τ_{alt} = 0.8 s for the 0.90 FRET state. **C)** Representative smFRET trace and idealized HaMMy trace for L14A TERT telomerase incubated with the $(TG)_8T_2G_3$ primer. **D)** Compiled histograms for L14A enzyme. L14A TERT demonstrated a dwell time of τ_{docked} = 0.4 s for the 0.50 FRET state and a dwell time of τ_{alt} =0.8 s for the 0.90 FRET state. As observed for U36-labeled enzyme, the L14A mutant demonstrates a strong effect on the docked state, but has a minimal effect on the alternative state.

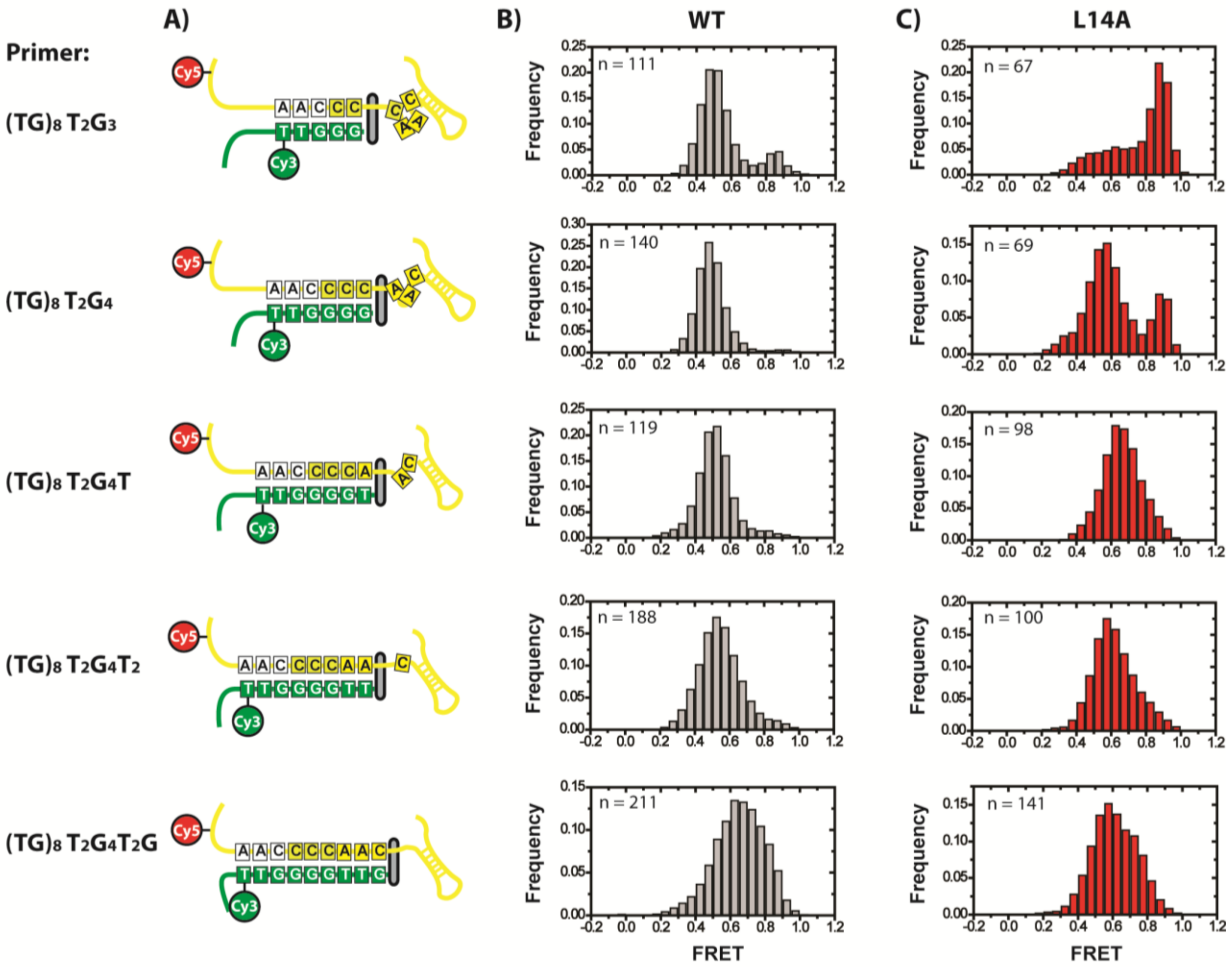

Figure S3. Effect of primer-template hybrid length on U63-labeled telomerase. Primers capable of forming 5-9 basepairs with template RNA were tested in smFRET telomerase binding assays with U63-labeled telomerase. **A)** Schematic diagram of the docked state for all six primers used in smFRET experiments, demonstrating the number of basepairs formed and positions of the Cy3 and Cy5 labels on the DNA primer and telomerase RNA, respectively. **B)** smFRET histograms for wild-type enzyme. As primers contain progressively more telomeric DNA sequence, the predominant FRET distribution of the docked state shifts from ~0.50 FRET to ~0.65 FRET. In addition, the ~0.90 FRET alternative state disappears. **C)** smFRET histograms for L14A mutant enzyme. The $(TG)_8T_2G_3$ primer is highly enriched for the alternative ~0.90 FRET state when compared against the wild-type enzyme. As the primer-template duplex is progressively extended, the L14A mutant FRET distribution increasingly resembles the wild-type distribution. The relative occupancies of the docked and alternative states in U63-labeled enzyme are highly consistent with the FRET distributions obtained with U36-labeled enzyme.

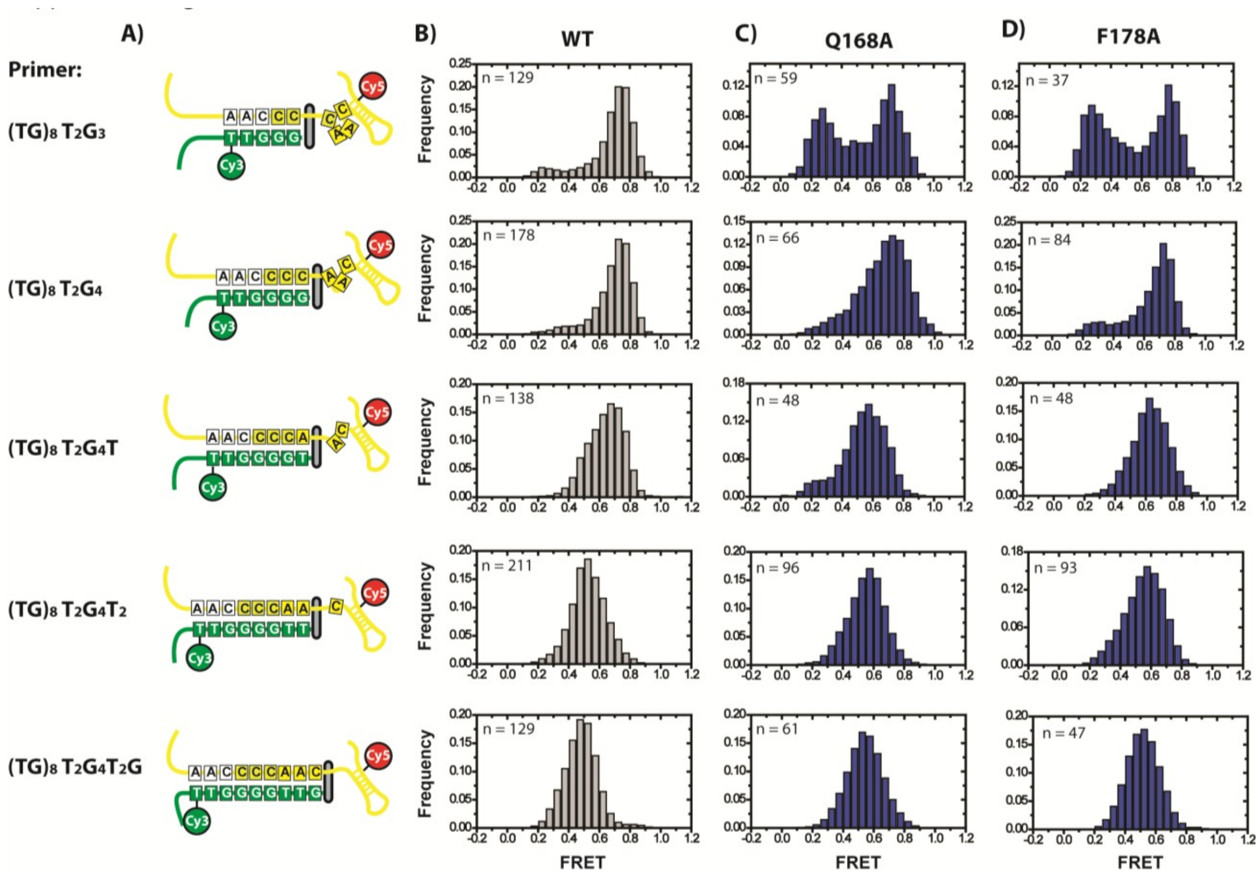

Figure S4. Effect of primer-template hybrid length on Q168A and F178A mutants. Primers capable of forming 5-9 basepairs with template RNA were tested in smFRET telomerase binding assays with U36-labeled telomerase. **A)** Schematic diagram of the docked state for all six primers used in smFRET experiments, demonstrating the number of basepairs formed and the expansion of the template RNA as the DNA-RNA duplex becomes progressively longer (2). **B)** smFRET histograms for wild-type enzyme. As primers contain progressively more telomeric DNA sequence, the predominant FRET distribution of the docked state shifts from ~ 0.75 FRET to ~ 0.50 FRET. In addition, the ~ 0.25 FRET alternative state disappears. **C)** smFRET histograms for Q168A mutant enzyme. As shown in Figure 2, Q168A mutants demonstrate an enrichment for the alternative state with the $(TG)_8T_2G_3$ primer. As the primer-template hybrid length becomes longer, the Q168A mutant increasingly resembles the wild-type distribution. **D)** smFRET histograms for F178A mutant enzyme. F178A mutants demonstrate similar trends to Q168A mutants in their FRET distributions.

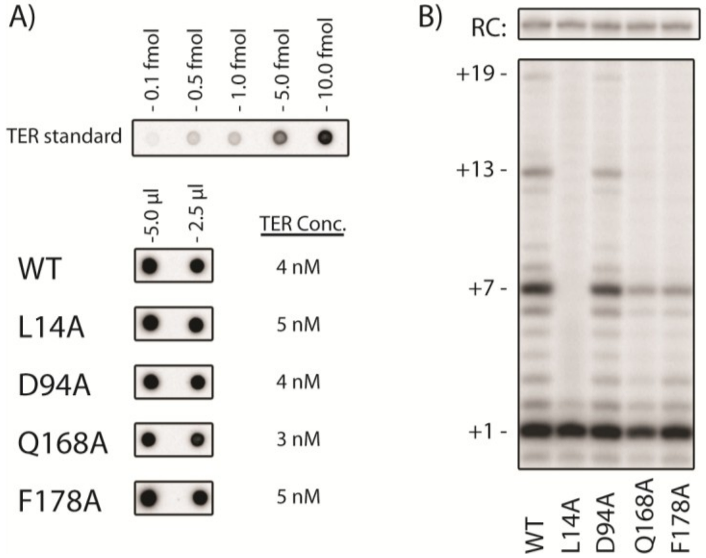

Figure S5. Quantification of immunopurified telomerase protein-RNA complexes. A) Wild-type and TEN domain mutant TERT was expressed in rabbit reticulocyte lysate and immunopurified by means of a FLAG tag on the N-terminus of TERT. Eluate from IP reactions was spotted on a blot and probed with a ^{32}P-labeled probe against TER to determine the concentrations of RNA that co-purified with TERT. 5 µl and 2.5 µl aliquots of the IP elution were compared against a TER standard. L14A, D94A, and F178A mutants demonstrated no detectible defect in the amount of TER that co-purified with TERT, whereas Q168A exhibited a modest defect in TER-TERT complexes. **B)** Telomerase primer extension assays on immunopurified telomerase. The material used in Figure S5A was used in telomerase primer extension assays using a $(GGGGTT)_3$ primer. As observed previously, L14A TERT exhibited a severe RAP defect and Q168A and F178A TERT also demonstrated RAP defects, whereas D94A telomerase activity largely resembled wild-type (3,4).

Supplementary Material references

1. McKinney, S.A., Joo, C. and Ha, T. (2006) Analysis of single-molecule FRET trajectories using hidden Markov modeling. *Biophys J*, **91**, 1941-1951.
2. Berman, A.J., Akiyama, B.M., Stone, M.D. and Cech, T.R. (2011) The RNA accordion model for template positioning by telomerase RNA during telomeric DNA synthesis. *Nat Struct Mol Biol*, **18**, 1371-1375.
3. Zaug, A.J., Podell, E.R. and Cech, T.R. (2008) Mutation in TERT separates processivity from anchor-site function. *Nat Struct Mol Biol*, **15**, 870-872.
4. Jacobs, S.A., Podell, E.R. and Cech, T.R. (2006) Crystal structure of the essential N-terminal domain of telomerase reverse transcriptase. *Nat Struct Mol Biol*, **13**, 218-225.
